# Physical Distancing Device with Edge Computing for COVID-19 (PADDIE-C19)

**DOI:** 10.3390/s22010279

**Published:** 2021-12-30

**Authors:** Chun Hoe Loke, Mohammed Sani Adam, Rosdiadee Nordin, Nor Fadzilah Abdullah, Asma Abu-Samah

**Affiliations:** Department of Electrical, Electronics and Systems Engineering, Faculty of Engineering and Built Environment, Universiti Kebangsaan Malaysia, Bangi 43600, Selangor, Malaysia; A166398@siswa.ukm.edu.my (C.H.L.); p106820@siswa.ukm.edu.my (M.S.A.); fadzilah.abdullah@ukm.edu.my (N.F.A.); asma@ukm.edu.my (A.A.-S.)

**Keywords:** COVID-19, computer vision, edge computing, thermometer, physical distancing

## Abstract

The most effective methods of preventing COVID-19 infection include maintaining physical distancing and wearing a face mask while in close contact with people in public places. However, densely populated areas have a greater incidence of COVID-19 dissemination, which is caused by people who do not comply with standard operating procedures (SOPs). This paper presents a prototype called PADDIE-C19 (Physical Distancing Device with Edge Computing for COVID-19) to implement the physical distancing monitoring based on a low-cost edge computing device. The PADDIE-C19 provides real-time results and responses, as well as notifications and warnings to anyone who violates the 1-m physical distance rule. In addition, PADDIE-C19 includes temperature screening using an MLX90614 thermometer and ultrasonic sensors to restrict the number of people on specified premises. The Neural Network Processor (KPU) in Grove Artificial Intelligence Hardware Attached on Top (AI HAT), an edge computing unit, is used to accelerate the neural network model on person detection and achieve up to 18 frames per second (FPS). The results show that the accuracy of person detection with Grove AI HAT could achieve 74.65% and the average absolute error between measured and actual physical distance is 8.95 cm. Furthermore, the accuracy of the MLX90614 thermometer is guaranteed to have less than 0.5 °C value difference from the more common Fluke 59 thermometer. Experimental results also proved that when cloud computing is compared to edge computing, the Grove AI HAT achieves the average performance of 18 FPS for a person detector (kmodel) with an average 56 ms execution time in different networks, regardless of the network connection type or speed.

## 1. Introduction

Public health and the global economy are under threat from the COVID-19 pandemic. As of 15 November 2021, there were 251 million confirmed cases and 5 million deaths from the COVID-19 outbreak [1]. Currently, the most effective infection prevention methods are physical distancing, wearing a face mask, and frequent handwashing [2]. The Malaysian government’s early response to the outbreak is to implement the Movement Control Order (MCO) at the national level to restrict the movement and gathering of people throughout the country, including social, cultural, and religious activities [3]. Besides that, government and private sectors cooperate in body temperature inspection and quarantine enforcement operations in all locations to prevent the spread of COVID-19. However, the critical issue is that it is not easy to implement strong and effective control measures on humans. People still need to address needs such as obtaining food from outside homes, working to cover living costs, and socializing with individuals or family members. The Ministry of Health Malaysia’s concern is that individuals do not take the standard operation procedures (SOP) compliance seriously and lack understanding of COVID-19 disease transmission [4]. In this context, intelligent and automated systems capable of operating 24 h a day to combat the pandemic transmission are critical for long-term economic and public health interests.

Edge computing is a concept that has been widely adopted in the healthcare industry to minimize the cost, energy, and workload of medical personnel [5,6]. Many types of Internet of Things (IoT) components have been proposed, including Radio Frequency Identification (RFID) and Bluetooth technology, magnetic field, infrared, camera and lidar sensors. These components play an important role in physical distancing monitoring via edge computing concept [7,8,9,10,11,12,13]. At the moment, physical distancing monitoring is primarily based on three technologies: wireless communication, electromagnetics, and computer vision. A Bluetooth-based wireless communication has been used to determine the distance between individuals based on the strength of the Received Signal Strength Indicator (RSSI) signal in [8]. Singapore has a “Tracetogether” application based on Bluetooth technology that enables close contacts of COVID-19 patients to be located [14]. The limitation of the application is that users have to download the existing application and then activate Bluetooth at all times in public places. Additionally, the most popular application of deep learning in this context is to detect physical distance using a camera [12]. However, such detection techniques are dependent on the camera location, computing power, and image processing capabilities.

Prevention is better than cure to break the chain of infection and combat the COVID-19 pandemic. The purpose of this study is to build a **P**hysic**a**l **D**istancing **D**ev**i**ce with **E**dge Computing for **C**OVID-**19** (PADDIE-C19) system to prevent infection based on the concept of edge computing. The three main functions of PADDIE-C19 are: (i) to identify and monitor physical distances using computer vision, (ii) forehead temperature checking, and (iii) to limit the number of people in each room or enclosed area through a counter system that detects people entering/leaving a premise. The edge computing unit can collect image and temperature data from sensors for processing at the edge without relying on the internet network. Furthermore, the edge computing system can provide real-time results and responses, as well as notifications and warnings to anyone who violates the 1-m physical distance rule or has a forehead temperature greater than 37.2 °C.

The following are the main contributions of this paper:

- A PADDIE-C19 prototype based on Raspberry-pi Grove Artificial Intelligence Hardware Attached on Top (Grove AI HAT) with edge computing capability to recognize and classify humans based on image processing. The performance of the person detector system implemented on the Grove AI HAT, Raspberry Pi 4 and Google Colab platform on different mobile networks was evaluated and compared based on frames per second (FPS) and execution time to compare the performance between edge and cloud computing approaches.
- A physical distance monitoring algorithm and implementation technique to operate in low-energy edge computing devices that provide physical distance guidance to the public.
- An accurate sensor platform design for forehead temperature measurement and person counting to manage the flow of visitors in public spaces.

The remainder of this research work is organized as follows. The problem background is demonstrated in Section 2 and the relevant works are discussed in Section 3. The PADDIE-C19 prototype physical distance monitoring algorithm and implementation technique with edge computing are comprehensively discussed in Section 4. The dataset used in this study for training and testing purposes is presented in Section 5. Furthermore, a detailed discussion and analysis on the FPS comparison between edge and cloud, execution time in different networks, the performance of person detector, distance test, comparison between the MLX90614 and Fluke 59 thermometer, and the person counter are discussed in this section. The concluding remarks and potential future research plans are provided in Section 6.

## 2. Problem Background

Many individuals are unaware or do not have the right knowledge of the seriousness of COVID-19 for individual health and its impact on society. On a cross-sectional survey to measure the level of awareness, views, and behaviours of the Malaysian public toward COVID-19, only 51.2% of participants were reported to wear face masks while out in public [15]. Inconsistent instructions and guidelines from the authorities also create feelings of panic and emotional stress, further reducing the society’s adherence to SOPs for pandemic prevention and physical distancing measures. Aside from psychological reasons, the cultural, economic, and geographical factors can create issues in addressing public violations with movement control orders enforcement. For example, in less than a week in December 2020, the police force in Selangor, Malaysia, issued 606 fines for those who failed to comply with the SOPs [16].

COVID-19 has changed the lives of the people since the MCO was implemented [15]. The government cannot permanently restrict people’s movement because it significantly influences the people’s way of life and the country’s economic sector. Although the flexibility of movement is given in the new life norms, SOPs often fail to be adhered to by the public, such as physical distancing, wearing a face mask, and registration process when entering the premises. Besides, MCO has placed high economic pressure on the low-income group. People require income from employment to meet their basic needs of food. Furthermore, the concept of working from home cannot be implemented in the manufacturing and service sectors. Moreover, large-scale gatherings such as religious activities and weddings can be considered to have serious cultural consequences if they are not conducted.

To fight the spreading of COVID-19, the Malaysian government has created an application, “MySejahtera” that records the entry of visitors into a facility [14]. However, a small percentage of citizens, particularly the elderly, do not own a smartphone. The application relies on QR code as user input, and is unusable for those without handphones. The application’s functionality is also limited to areas with consistent internet coverage. Furthermore, the government and employers are experiencing a shortage of officers and staff responsible for ensuring that people are physically separated at least one meter in public places at all times.

This article aims to create a COVID-19 monitoring system based on the concepts of edge computing, computer vision and the Internet of Things. An automated monitoring system called PADDIE-C19 has been designed to maintain the recommended safe physical distance between crowds in factories, schools, restaurants, and ceremonies to confine the spread of COVID-19. A person detection model is trained based on the transfer learning method and is used to measure physical distances via camera. Meanwhile, an infrared thermometer is used to detect the individual’s forehead temperature at the entrance to identify people with symptoms of COVID-19 infection. Last but not least, the number of people in any room or enclosed location is also limited by using a visitor counting system based on an ultrasonic sensor.

## 3. Related Works

The COVID-19 pandemic has impacted hospitals worldwide, causing many non-emergency services and treatments, such as cancer, hypertension, and diabetes, to be delayed [17]. This is due to a shortage of healthcare workers to handle COVID-19 patients. Nevertheless, medical equipment with edge computing capability for patient monitoring can help to reduce the load on medical systems. Several studies in Table 1 demonstrate the role of edge computing in healthcare and the COVID-19 pandemic.

According to a current study on edge computing, physical distancing application solutions are divided into three types of technologies: wireless communication, electromagnetics, and computer vision. Therefore, related sensors such as RFID, Bluetooth, magnetic fields, infrared sensors, cameras, and lidars are currently used in existing systems for people detection and physical distancing detection. Each of the following technologies has strengths and weaknesses, which are summarized in Table 2.

Object detection based on neural networks and deep learning is the basis of computer vision algorithms to perform person detection in an image or video frame. Computer vision technology with an RGB camera, infrared camera and lidar sensors is widely used in physical distancing monitoring. Table 3 shows some examples of approaches in physical distancing monitoring using computer vision.

## 4. Methodology

### 4.1. PADDIE-C19 System’s Flow Chart

**Figure 1:**
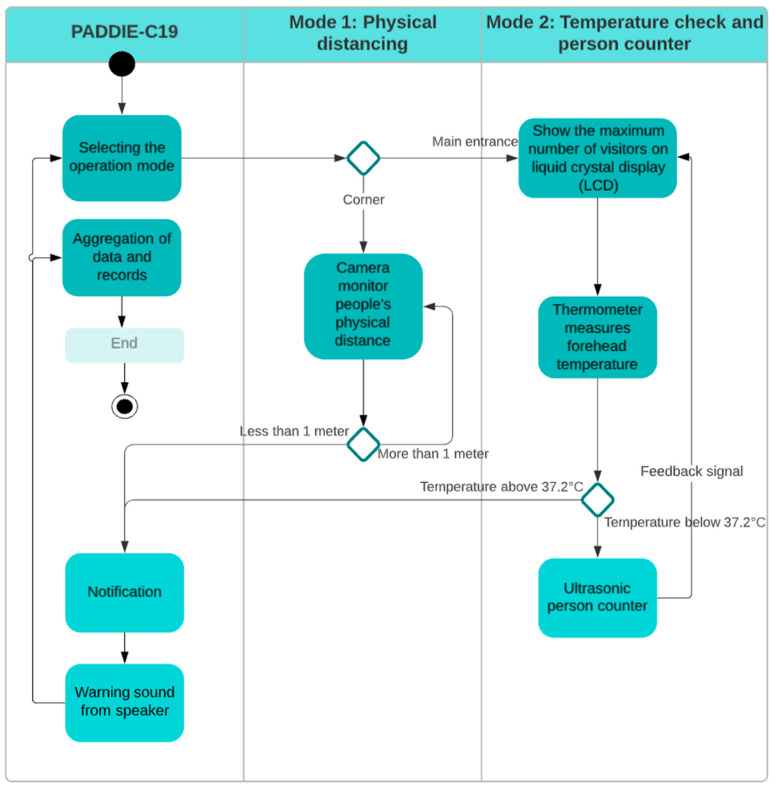
The proposed PADDIE-C19 system’s flow chart.

Figure 1 illustrates the flow of PADDIE-C19 operations at the edge, where computation and data storage are located closer to the primary user. PADDIE-C19 operates in two modes: (i) physical distancing monitoring, and (ii) temperature measurement with a person counter. PADDIE-C19 will be in the first mode if installed at the viewpoint corner. Grove AI HAT equipped with an RGB camera is used to monitor physical distancing compliance. A loudspeaker will deliver a warning sound when individuals fail to maintain a physical distance of at least one meter. After that, in the second mode of operation, the infrared thermometer will take the forehead temperature of each individual without making contact before allowing them to enter the building or enclosed area. Simultaneously, the ultrasonic sensor will detect anyone passing through the main door. The Raspberry Pi will be responsible for recording and processing temperature data and people counter data. Following that, both measured values will be displayed on the LCD, along with the maximum number of people permitted in a room or area.

### 4.2. PADDIE-C19 Block Diagram

**Figure 2:**
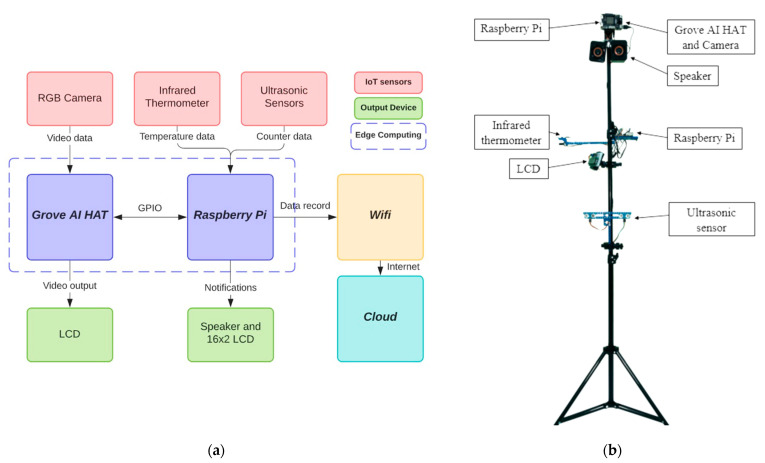
(**a**) Block diagram of PADDIE-C19 system and (**b**) PADDIE-C19 system prototype.

The PADDIE-C19 prototype is illustrated in Figure 2b. A 2 megapixels OV2640 RGB camera is used to provide video data to Grove AI HAT, the edge computing unit for people tracking and physical distance monitoring. An LCD with a resolution of 320 × 240 is used to display the detection results while running the program. The OV2640 camera and LCD connect to the Grove AI HAT via a 24-pin connector with a serial communication protocol. Two ultrasonic and one infrared sensor are connected to the Raspberry Pi microcontroller. A speaker is connected to the analog audio output of the Raspberry Pi to provide warning sound if there are individuals who do not comply with the physical distance rules. The Grove AI HAT and Raspberry Pi can be powered by a 5 V/2 A power adapter via a USB connector. The Raspberry Pi is connected to the internet and eventually the cloud via Wi-Fi, and can be remotely controlled via the VNC Connect software. The PADDIE-C19 design concept is based on the installation at indoor public locations such as shops, offices, and factories. All the connections can be referred to in Figure 2a.

**Figure 3:**
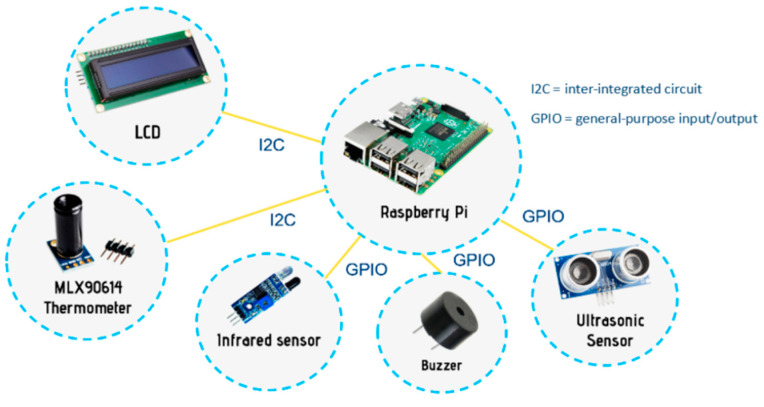
Connection of the Raspberry Pi to sensors and output devices.

Figure 3 shows the setup of Raspberry Pi with an MLX90641 infrared thermometer used to measure forehead temperature whenever a user places his head in front of it. The forehead distance from the infrared thermometer can be calculated using an infrared sensor. This is due to the fact that each type of thermometer has a unique distance-to-spot ratio. As a result, temperature taking is permitted only at a distance of 3 to 5 cm for the MLX90614 thermometer to obtain an accurate forehead temperature reading. The infrared camera connects to the Raspberry Pi via the I2C protocol. The buzzer will emit a signal sound when the temperature is successfully measured. Furthermore, two ultrasonic sensors were used to detect people moving in and out of the premises from a distance. If the first sensor detects the obstacle ahead of time, the number of people recorded will be increased by one. The recorded number of people will be reduced by one if the second sensor detects the obstacle ahead of time. Algorithm 1 describes the physical distancing monitoring system that consists of two functions.
**Algorithm 1.** Physical Distancing Monitoring.**Input:** V_n_: Video V containing N number of frames of size 160*160/[0P,1P,2P] 224*224/[0P,1P,2P] **Output:** D: Safe and unsafe Distance vector between two objects **Initialize Parameter:** Distance_Threshold = 100 cm, Temp_Threshold = 37.2, Visitor_Count = 0, Max_Visitor = 15, **Function1** Physical distancing () Select = human_detection_framework For () in range (Human_Count) // person detection for each frame in video   For x in range(x): // number of person more than 1     D = √((x_2 − x_1)^2 + (y_2 − y_1)^2     // calculate constant, k = (actual distance, cm)/(pixel distance)          If D <= Distance_Threshod: // less than 1 m               Send notification // output from speaker          **EndIF**     **Endfor** **Endfor** **EndFunction1** **Function2** Temperature check and person counter For number of Visitor_Count <= Max_Visitor,           Show max number of visitors      For (temp_Threshold < 37.2) in range (Visitor_Count)             For x in range (x):                 If proximity sensor detected object at 3 cm distance                 // calculate forehead temperature                        **if** Temp_Threshold < 37.2                              Pass                        **Else**                             Display: fever no entry                        **EndIF**                       **EndIF**               **EndFor**     **EndFor** **EndFunction2**

### 4.3. Physical Distancing Implementation Steps

**Figure 4:**
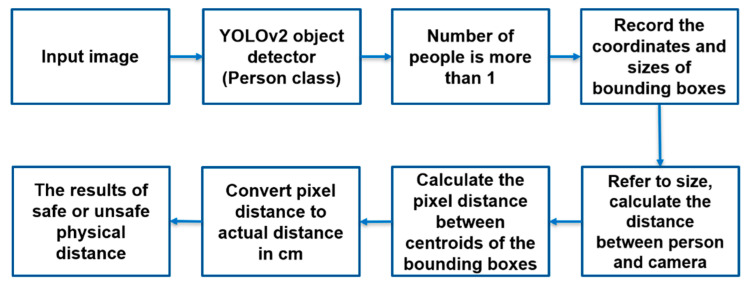
Physical distancing flow chart.

The Grove AI HAT is based on the Sipeed Maix M1 AI module and the Kendryte K210 processor, which is capable of running person detection models for physical distancing applications. The general-purpose neural network processor or the KPU inside the Grove AI HAT can accelerate the convolutional neural network (CNN) model calculations with minimal energy. Kendryte K210 KPU only recognizes models in kmodel format based on the YOLOv2 object detector. The basic steps involved in in-person detection and physical distance measurement in Grove AI HAT are shown in Figure 4.

In this paper, the person detector based on the YOLOv2 model [24] with MobileNet backbone was trained using transfer learning with Tensorflow framework on an Ubuntu 18.04 machine equipped with an Nvidia GTX 1060. A total of 5632 images were collected and downloaded from Kaggle, CUHK Person, and Google Images from the open-source datasets platform, which the details are summarized in Table 4. Following that, all images were converted to JPEG format with a 224 × 224 resolution. With LabelImg software, all data images were manually labelled with a bounding box as objects of interest in the “Person” class. The original MobileNet weights file was loaded into the Ubuntu machine, along with the processed dataset, and trained until the validation loss curve stopped improving. Once the training is complete, the tflite file will be generated along with the most recent updated weights file. Tensorflow lite files need to be converted to kmodel format via the NNCase Converter tool so that a trained neural network can be run at KPU Grove AI HAT for person detection.

The YOLOv2-based person detection model was used to detect people from images captured from the OV2640 camera. Next, the KPU Grove AI HAT obtains the information of the people’s sizes and coordinates detected in the bounding boxes. The distance between two detected persons was then calculated according to the centroid of the bounding boxes. The estimated physical distance between individuals was determined using the pixel distance on the LCD. Equation (1) shows the distance formula, with *d* used to find the pixel distance between two coordinates of the centroid using Pythagoras’ theorem. If the bounding box fails to keep a minimum distance of one meter from the others, it will turn red.

**Figure 5:**
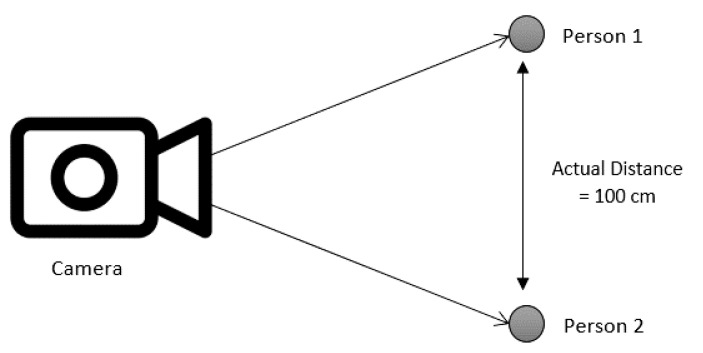
Determination of actual distance from the pixel distance at a fixed camera distance.

With reference to [22] and Figure 5, the actual distance in centimeters can be calculated according to how many pixels have been used in 1 m with a directly proportional formula, *k*, as shown in Equation (2). While it is obvious that the rate of change of pixel distance is directly proportional to the actual distance, this method requires calibration according to a fixed camera distance. To improve the accuracy of distance determination, three constant values must be determined with an actual distance of 100 cm between two people using three tests at camera distances of 200 cm, 300 cm, and 400 cm. Since the camera is installed at a fixed location to detect a small area of people, the distance between the camera and people is considered constant. Multiple PADDIE-C19 devices can be arranged in multiple places to cover a larger crowd for detection

(1) $$pixel\;distance,\;d=\sqrt{{\left(x_{2}-x_{1}\right)}^{2}+{\left(y_{2}-y_{1}\right)}^{2}}$$

(2) $$Proportional\;distance,\;k=\frac{actual\;distance,cm}{pixel\;distance}$$

### 4.4. System Evaluation Metrics

In this study, execution time, in seconds, is considered as a metric to determine edge computing performance in real-time implementations. Comparison of execution time based on Wi-Fi, 4G, 3G and 2G networks were implemented between the Raspberry Pi 4, Grove AI HAT and Google Colab platforms. Besides, the effectiveness of the object detection model can be measured with the FPS of output video on the LCD. To examine the benefits of deploying object detection and recognition on edge computing over cloud computing, a YOLOv4 model [25] was trained in Google Colab using the same dataset from Grove AI HAT. Then, the YOLOv4 model was run on the Raspberry Pi 4 and Google Colab platforms to compare frames per second and execution time with the Grove AI HAT using the kmodel shown in Table 5. In addition, the confusion matrix can be used to compute the precision of the trained person detection model. Furthermore, the algorithm for determining physical distance can be tested for accuracy by performing a practical in front of the camera and comparing the measured distance from the pixels with the actual distance using a tape measure.

## 5. Results and Discussions

### 5.1. FPS Comparison between Edge and Cloud

**Figure 6:**
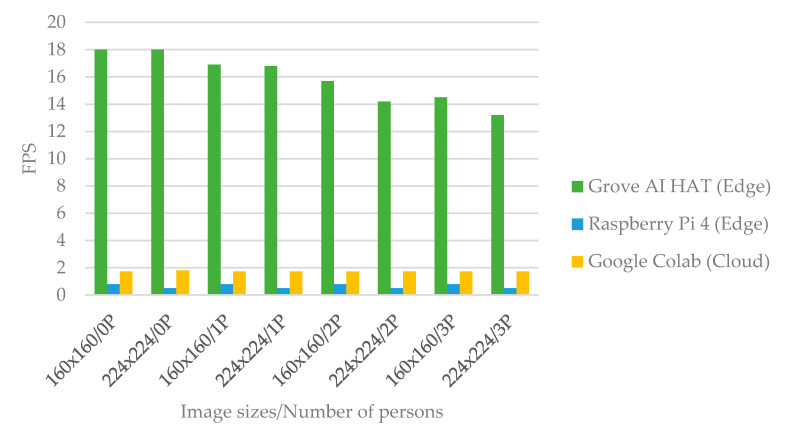
FPS comparison of three platforms based on image size and the number of people.

The object detection model is evaluated in terms of FPS to determine how fast the video is processed in Grove AI HAT to detect the object of interest. The central processor unit (CPU) is a significant contribution to frame rate. To examine the differences between edge computing and cloud computing, a YOLOv2-based person detection model was run on Grove AI HAT, and a YOLOv4-based person detection model was run on Raspberry Pi and Google Colab. Due to hardware and software limitations, the YOLOv4 model could not be run on the Grove AI HAT because the kmodel detection model is dedicated for use with the KPU Grove AI HAT. Figure 6 depicts the change of FPS between the three platforms based on the size of the input image and the number of people. Grove AI HAT achieves a maximum FPS of 18 FPS on the LCD, but begins to degrade as the number of people standing in front of the camera increases. Based on the same image size input, Google Colab and Raspberry Pi cannot achieve more than 2 FPS. Google Colab is GPU-enabled. However, the GPU detection resulted in a considerable live streaming delay due to the limited bandwidth of the chosen networks. Finally, the Grove AI HAT on edge outperforms the Raspberry Pi on edge and Google Colab on the cloud in terms of video smoothness.

### 5.2. Execution Time in Different Networks

**Figure 7:**
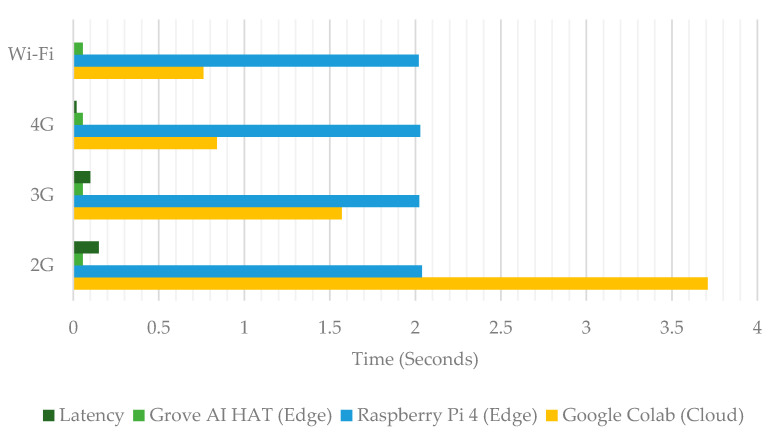
Experimental program execution time in different networks.

Table 6 displays the evaluation of a Python script’s execution time to complete one iteration in four network conditions. Chrome DevTools was used to define Wi-Fi, 4G, 3G, and 2G internet network profiles to monitor and control network activity. Figure 7 shows that the time spent by Google Colab increases as the Internet speed decreases from the 4G network to the 2G network. However, the execution time of Python scripts on edge computing device terminals remains the same because video data processing services do not rely on the internet. This is due to the fact that Google Colab runs an object detection model in the cloud and its performance and stability are highly dependent on the internet network’s stability and speed. A large amount of internet bandwidth is required to upload video data before the person detection process is carried out in the cloud.

### 5.3. Performance of Person Detector

The confusion matrix was used to evaluate the person detection model’s efficiency in Grove AI HAT and Google Colab. The detection model was trained to predict the “person” class and generate a bounding box to the detected person in the LCD. According to Table 7 and Table 8, Google Colab achieves 95.45% accuracy in person classification, whereas Grove AI HAT achieves only 74.65% accuracy. Grove AI HAT does not detect people from a far distance due to memory constraints and the KPU in Grove AI HAT only allows a 224 × 224 image input resolution from the camera. Moreover, the kmodel neural network file size loaded into Grove AI HAT is 1.8 MB, less than the 202 MB file weights used in YOLOv4. Because of their low hardware performance requirements, small neural networks are ideal for edge devices, but they can also produce lower accuracy values.

### 5.4. Distance Test

**Figure 8:**
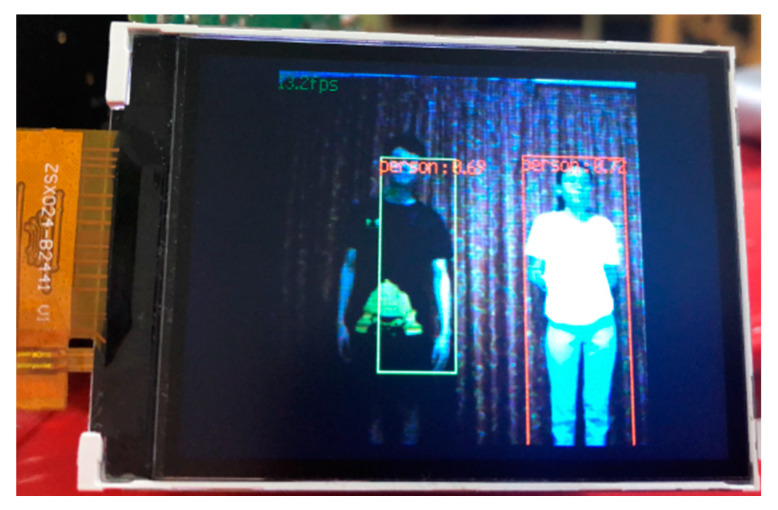
Pixel distance measured from the LCD of Grove AI HAT.

**Figure 9:**
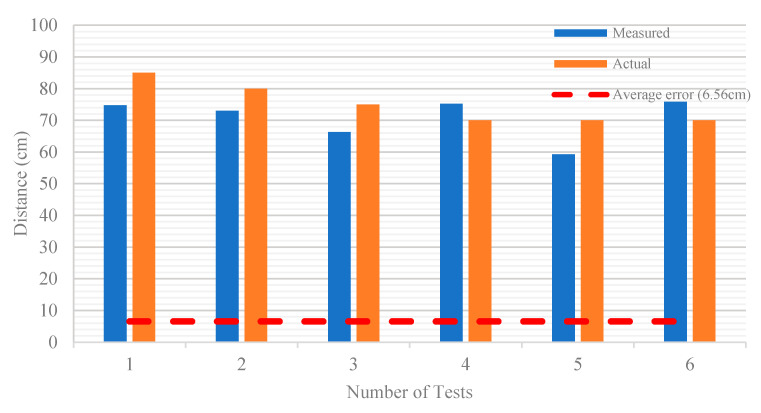
Measured versus actual distance of two people and the average error.

The size and coordinates of the boundary box generated by the KPU Grove AI HAT on the detected person can be used to calculate the physical distance between them. The method of measuring the distance between two people in Grove AI HAT is first to calculate the pixel distance, *d* between two people in the image using Equation (1) and then convert the distance into centimeter using constant *k* in Equation (2). Figure 8 shows an example of physical distance measured from the LCD and Table 9 lists the constant *k* values according to camera distance. Figure 9 summarizes the measured distance from the pixels compared to the actual distance measured from the measuring tape. The average error between the measured and actual values is shown in red dotted line and the mean absolute error (MAE) between them is 8.95 cm. However, the disadvantage of this method is that the detected person’s measurement and height will affect the accuracy of the distance measurement between two people. Additionally, Grove AI HAT operations such as person detection and physical distance monitoring can only be performed from a frontal view. The 224 × 224 image input resolution was insufficient to produce high-quality person detection at a long-range distance. The main limitation is that the KPU Grove AI HAT has a limited memory capacity, making it incapable of handling image input resolutions greater than 224 × 224 from the camera.

### 5.5. Comparison between MLX90614 and Fluke 59 Thermometer

**Figure 10:**
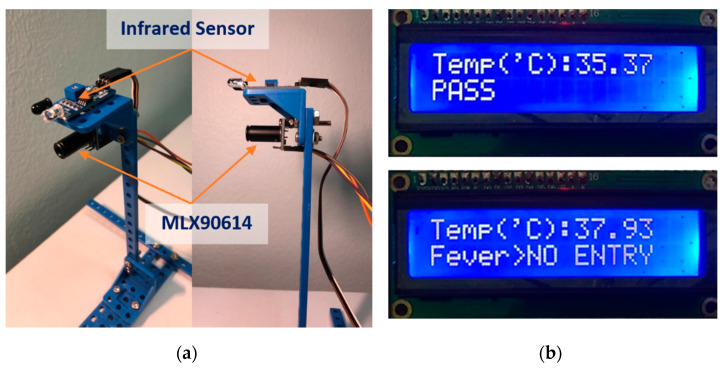
(**a**) Forehead MLX90614 thermometer prototype and (**b**) LCD displays either pass or no entry based on detected temperature with a limit of 37 °C.

To test the performance of the MLX90614 infrared thermometer, a comparison was made with the manual Fluke 59 thermometer that is widely available for consumers. The forehead temperature measurement prototype is made up of an infrared sensor for obstacle detection and an MLX90614 thermometer (Figure 10a). When the infrared sensor detects an obstacle within 3 cm from the MLX90614 thermometer, it will collect temperature data. The MLX90614 sensor will convert the infrared radiation signal collected from the forehead into electrical signals, which will then be processed by Raspberry and displayed temperature value on the LCD. When a person’s temperature is detected above 37.2 °C, the message “FEVER > NO ENTRY” appears on the LCD immediately, as shown in Figure 10b.

**Figure 11:**
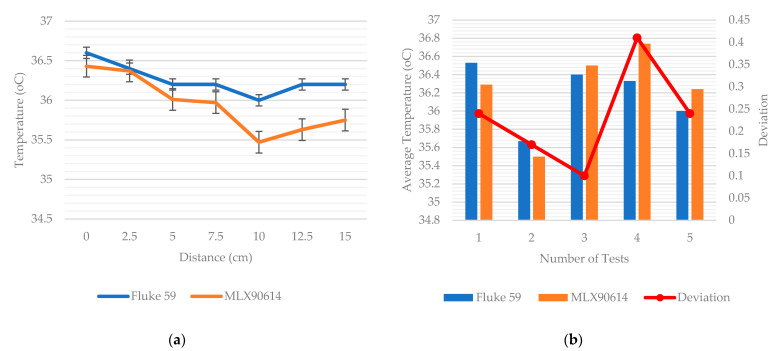
(**a**) The temperature of the forehead from various distances using Fluke 59 and MLX90614; (**b**) average temperature with Fluke 59 and MLX90614 fixed at a distance of 3 cm.

The graph in Figure 11a illustrates the values of forehead temperature obtained from Fluke 59 and MLX90614 at various distances ranging from 0 cm to 15 cm. The temperature value of the MLX90614 thermometer gradually decreases after 5 cm due to the lower distance to spot ratio compared to the Fluke 59 thermometer. According to the product specifications, MLX90614 has a 1.25 distance to spot ratio, whereas Fluke 59 has a value of 8. In addition, the standard deviation value of the MLX90614 is 0.3346, which is higher than the value of 0.1761 for Fluke 59. When the measuring distance exceeds 5 cm, taking the temperature value from MLX90614 becomes less accurate.

However, the MLX90614 performs well when measuring temperature from a distance of 3 cm. To obtain deviation values between the two types of temperature sensors, measurements were repeated three times over five people. Figure 11b shows the forehead temperature data obtained at a distance of 3 cm with the Fluke 59 and MLX90614 thermometers, and the temperature difference between the two thermometers was only 0.1 to 0.4 °C.

### 5.6. Person Counter

**Figure 12:**
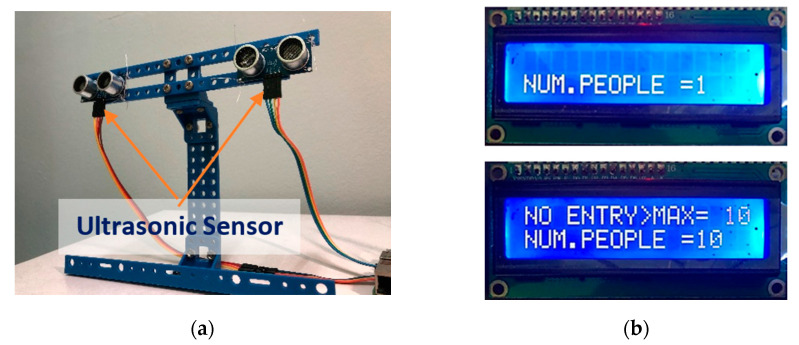
(**a**) Person counter prototype and (**b**) LCD indicates the number of people equal to 1 and the no entry indication if there are more than 10 individuals.

The person counter prototype is shown in Figure 12a, which consists of two ultrasonic sensors to add confidence to the readings. It will be installed at the premises’ main entrance and will count people’s movements bidirectionally. However, the current system limitation is the capability of counting only one person passing through the sensor at a time. The refresh rate of the two ultrasonic sensors in detecting passing people is 9.8 Hz. If someone passes from left to right, the number of people will be added by one on the LCD, while if someone passes from right to left, the number of people will be subtracted by one. If the number exceeds the maximum limit, the LCD will indicate no entry as shown in Figure 12b. Note that the maximum limit can be reconfigured according to the preference of the premise’s requirement.

### 5.7. Summary of PADDIE-C19 Performance

To achieve the study’s objective, the design of the PADDIE-C19 prototype has been developed in the alpha stage, i.e., the early design process testing. Each of its features, employed evaluation metrics and experimental results are summarized in Table 10. Issues of poor detection accuracy have been identified and the design will be refined with upgraded hardware to fulfill the needs of a real-world application. The systematic error of 0.5 provided the balance for capturing two different temperatures (Forehead and environment).

## 6. Conclusions

The study proposed an edge computing prototype to monitor physical distancing that measures the forehead temperature and keeps track of the person count in managing the flow of visitors in the public spaces. The PADDIE-C19 prototype has a small and portable design for temperature screening and person counting applications. The Grove AI HAT edge computing device on PADDIE-C19 was proven to have a higher frame rate per second than the cloud-based Google Colab and Raspberry Pi, but the accuracy of in-person tracking is relatively lower. While Grove AI HAT can perform all computation at the edge, the main advantage of the Raspberry Pi-based system is that it can be controlled remotely via the internet using VNC software. All hardware only requires a 5 V power supply, which gives the energy saver benefit compared to other commercial devices. Further study might be conducted to solve PADDIE-C19’s shortcomings, such as frontal view-only detection, by replacing Grove AI HAT with edge computing devices that come with bigger memory and higher computing capabilities, such as the Nvidia Jetson Nano and LattePanda Alpha. This way, the accuracy of the person detector can be increased by running larger neural network models at the edge computing device. The second recommendation is to install better resolution cameras to improve the accuracy of person detection from a long distance. Finally, the PADDIE-C19 system can be improved by including a global positioning system (GPS) for outdoors, or Wifi/RFID/Bluetooth-based localization for indoors, to determine the exact location of each PADDIE-C19 system based on various potential locations for public health monitoring in the age of a new normal.

## Figures and Tables

**Table 1:** Studies on edge computing in the area of healthcare.

References	Contribution
[18]	A real-time patient monitoring system that reduces energy usage, data upload cost and delay between sensor transmission and reception.
[19]	Proposed for health services and mobile edge computing (MEC) to deliver augmented reality (AR)-based remote surgery with latency in microseconds and bandwidth over 30 Gbps.
[20]	Edge-computing system that detects fever and cyanosis to relieve staff overload. The developmental test results showed a 97% accuracy in detecting fever and 77% in detecting cyanosis.

**Table 2:** Comparison of existing physical distancing solutions.

Technology	Hardware	Advantage	Limitation
Wireless communication	Radio frequency identification (RFID) [7]	- Quick response within 1 s - $1.95 cost per unit	Body contacts detection but does not offer accurate distance measurement between users
	Bluetooth [8]	- Real-time physical distance warning with over 80% accuracy	Detection relies on the person who installs the app, but the battery drains quickly
Electromagnetic	Magnetic field [9]	- Capable of detecting objects at a distance of 2 m without interference	The device is large and not portable
	Passive infrared [10]	- 240° detection angle with physical distance alerts	Obstacles easily disrupt the infrared rays
Computer vision	Camera and Lidar [11]	- Robots can identify and track individuals who fail to keep a physical distance	Unable to distinguish between family members and strangers
	Camera [12]	- The system achieves an average accuracy of 99.8% with 24.1 frames per second (FPS)	The location of the camera affects the detection accuracy

**Table 3:** Physical distancing solutions based on computer vision.

References	Method	Result	Limitation
[21]	Thermal cameras and Nvidia Jetson Nano are used to monitor people’s physical distances.	The object detector with Dataset I achieves 95.6% accuracy and 27 FPS with the proposed approach.	There is no temperature screening for fever individuals.
[22]	Individuals’ physical distances are monitored using a ToF (time-of-flight) camera and the YOLOv4 model.	The suggested model’s mAP (mean average precision) score is 97.84% and the MAE (mean absolute error) between real and measured physical distance is 1.01 cm.	Experiments were carried out with the Tesla T4 graphics processing unit (GPU), which has large power consumption and is not portable.
[23]	Automatic patrol robots that monitor people’s physical distances and face masks.	A patrol robot equipped with a camera and speaker to promote physical distancing and mask wearing.	Not suitable for use in small spaces or indoors.

**Table 4:** Datasets description.

Class	Sources	Size	Description
Person	Kaggle Dataset	785	A person was walking on the road.
	CUHK Person Dataset	3840	Walking pedestrians at a various angle.
	Google Open Images	1007	Randomly sampled person from different backgrounds.

**Table 5:** Comparison of detection models between edge and cloud platforms.

	Raspberry Pi 4 (Edge)	Grove AI HAT (Edge)	Google Colab (Cloud)
Processor	ARM Cortex-72	M1 K210 RISC-V	Dual Intel Xeon
Clock (GHz)	1.5	0.4–0.6	2.2
RAM (GB)	4	0.008	13.3
AI Resources	-	KPU	Tesla T4
Language	Python	MicroPython	Python
Model	YOLOv4	kmodel	YOLOv4

**Table 6:** Internet profiles, based on several network technologies.

Network	Download	Upload	Latency
Wi-Fi	30 Mbps	15 Mbps	5 ms
4G	4 Mbps	3 Mbps	20 ms
3G	750 kbps	250 kbps	100 ms
2G	200 kbps	100 kbps	150 ms

**Table 7:** Accuracy of the person detector with Grove AI HAT.

Grove AI HAT Confusion Matrix				
		Predicted Class		
		Person	No person	**Recall**
**Actual class**	Person	32	12	0.7273
	No person	6	21	0.7778
	**Precision**	0.8421	0.6364	**Accuracy = 0.7465**

**Table 8:** Accuracy of the person detector with Google Colab.

Google Colab Confusion Matrix				
		Predicted Class		
		Person	No person	**Recall**
**Actual class**	Person	38	1	0.9744
	No person	2	25	0.9259
	**Precision**	0.95	0.9615	**Accuracy = 0.9545**

**Table 9:** Constant values determined at different camera distances.

Actual Physical Distance	Camera Distance	Pixel	Constant, *k*
100 cm	200 cm	127	0.7874
100 cm	300 cm	101	0.9901
100 cm	400 cm	54	1.8519

**Table 10:** Summary of the performance of various devices in PADDIE-C19.

Feature	Evaluation Metrics	Experimental Result
Grove AI HAT with edge computing	Frame per second (FPS)	Grove AI HAT achieves the average performance of 18 FPS with a person detector (kmodel).
Average execution time	Second (s)	The average execution time is 56 ms in different networks.
Person detector	Classifier accuracy	The accuracy of kmodel to distinguish person class is 74.65%.
Physical distancing	Centimeter (cm)	The average absolute in measuring distance is 8.95 cm.
MLX90614 Thermometer	Celsius (°C)	The systematic error in measuring forehead and ambient temperatures is less than 0.5 °C.
Person counter	Hertz (Hz)	The refresh rate in detecting a person is 9.8 Hz.

## Data Availability

The data presented in this study are openly available in our GitHub (Link: https://github.com/Ya-abba/COVID-19-Physical-Distancing-Monitoring-Device-with-the-Edge-Computing.git) and Google Drive repository (Link: https://drive.google.com/file/d/1zcESizKClTiKIyl64w7D0SbIr3_7vf2g/view?usp=sharing) (accessed on 7 October 2021).

## References

[1] Law T. (2021). 2 Million People Have Died From COVID-19 Worldwide. Time.

[2] WHO (2021). Coronavirus Disease (COVID-19) Advice for the Public.

[3] Shah A.U.M., Safri S.N.A., Thevadas R., Noordin N.K., Rahman A.A., Sekawi Z., Ideris A., Sultan M.T.H. (2020). COVID-19 outbreak in Malaysia: Actions taken by the Malaysian government. Int. J. Infect. Dis..

[4] Abdali T.-A.N., Hassan R., Aman A.H.M. (2021). A new feature in mysejahtera application to monitoring the spread of COVID-19 using fog computing. Proceedings of the 2021 3rd International Cyber Resilience Conference (CRC).

[5] Albayati A., Abdullah N.F., Abu-Samah A., Mutlag A.H., Nordin R. (2020). A Serverless Advanced Metering Infrastructure Based on Fog-Edge Computing for a Smart Grid: A Comparison Study for Energy Sector in Iraq. Energies.

[6] Abdali T.-A.N., Hassan R., Aman A.H.M., Nguyen Q.N. (2021). Fog Computing Advancement: Concept, Architecture, Applications, Advantages, and Open Issues. IEEE Access.

[7] Garg L., Chukwu E., Nasser N., Chakraborty C., Garg G. (2020). Anonymity Preserving IoT-Based COVID-19 and Other Infectious Disease Contact Tracing Model. IEEE Access.

[8] Ng P.C., Spachos P., Plataniotis K.N. (2021). COVID-19 and Your Smartphone: BLE-based Smart Contact Tracing. IEEE Syst. J..

[9] Bian S., Zhou B., Lukowicz P. (2020). Social distance monitor with a wearable magnetic field proximity sensor. Sensors.

[10] Nadikattu R.R., Mohammad S.M., Whig P. (2020). Novel economical social distancing smart device for covid19. Int. J. Electr. Eng. Technol..

[11] Sathyamoorthy A.J., Patel U., Savle Y.A., Paul M., Manocha D. (2020). COVID-Robot: Monitoring social distancing constraints in crowded scenarios. arXiv.

[12] Rezaei M., Azarmi M. (2020). Deepsocial: Social distancing monitoring and infection risk assessment in covid-19 pandemic. Appl. Sci..

[13] Nguyen C.T., Saputra Y.M., Van Huynh N., Nguyen N.T., Khoa T.V., Tuan B.M., Nguyen D.N., Hoang D.T., Vu T.X., Dutkiewicz E. (2020). A Comprehensive Survey of Enabling and Emerging Technologies for Social Distancing—Part I: Fundamentals and Enabling Technologies. IEEE Access.

[14] Goggin G. (2020). COVID-19 apps in Singapore and Australia: Reimagining healthy nations with digital technology. Media Int. Aust..

[15] Azlan A.A., Hamzah M.R., Sern T.J., Ayub S.H., Mohamad E. (2020). Public knowledge, attitudes and practices towards COVID-19: A cross-sectional study in Malaysia. PLoS ONE.

[16] Idris M.N.M. (2020). 606 Kompaun Langgar SOP di Selangor. Utusan Malaysia.

[17] WHO (2020). COVID-19 Significantly Impacts Health Services for Noncommunicable Diseases.

[18] Mohsin J., Saleh F.H., Al-muqarm A.M.A. (2020). Real-time Surveillance System to detect and analyzers the Suspects of COVID-19 patients by using IoT under edge computing techniques (RS-SYS). Proceedings of the 2020 2nd Al-Noor International Conference for Science and Technology (NICST).

[19] Ranaweera P.S., Liyanage M., Jurcut A.D. (2020). Novel MEC based Approaches for Smart Hospitals to Combat COVID-19 Pandemic. IEEE Consum. Electron. Mag..

[20] Hegde C., Jiang Z., Suresha P.B., Zelko J., Seyedi S., Smith M., Wright D., Kamaleswaran R., Reyna M., Clifford G. (2020). AutoTriage—An Open Source Edge Computing Raspberry Pi-based Clinical Screening System. medRxiv.

[21] Saponara S., Elhanashi A., Gagliardi A. (2021). Implementing a real-time, AI-based, people detection and social distancing measuring system for Covid-19. J. Real-Time Image Process..

[22] Rahim A., Maqbool A., Rana T. (2021). Monitoring social distancing under various low light conditions with deep learning and a single motionless time of flight camera. PLoS ONE.

[23] Shen Y., Guo D., Long F., Mateos L.A., Ding H., Xiu Z., Hellman R.B., King A., Chen S., Zhang C. (2021). Robots under COVID-19 Pandemic: A Comprehensive Survey. IEEE Access.

[24] Redmon J., Farhadi A. YOLO9000: Better, faster, stronger. Proceedings of the IEEE Conference on Computer Vision and Pattern Recognition.

[25] Bochkovskiy A., Wang C.-Y., Liao H.-Y.M. (2020). Yolov4: Optimal speed and accuracy of object detection. arXiv.

